# Characterization of Two Novel Predatory Bacteria, *Bacteriovorax stolpii* HI3 and *Myxococcus* sp. MH1, Isolated from a Freshwater Pond: Prey Range, and Predatory Dynamics and Efficiency

**DOI:** 10.3390/microorganisms10091816

**Published:** 2022-09-10

**Authors:** Daisuke Inoue, Naoto Hiroshima, So Nakamura, Hidehiro Ishizawa, Michihiko Ike

**Affiliations:** 1 Division of Sustainable Energy and Environmental Engineering, Osaka University, 2-1 Yamadaoka, Suita 565-0871, Japan; 2 Department of Applied Chemistry, University of Hyogo, 2167 Shosha, Himeji 671-2280, Japan

**Keywords:** *Bacteriovorax*, *Myxococcus*, predatory bacteria, isolation, freshwater pond, predation dynamics, prey range

## Abstract

Predatory bacteria, which prey on other bacteria, have significant functions in microbial ecosystems and have attracted increasing attention for their biotechnological use. However, knowledge of the characteristics of wild-type environmental predatory bacteria remains limited. This study isolated two predatory bacteria, *Bacteriovorax stolpii* HI3 and *Myxococcus* sp. MH1, from a freshwater pond and characterized their predation capabilities. Determination of the prey range using 53 potential prey strains, including 52 environmental strains, revealed that *B. stolpii* HI3 and *Myxococcus* sp. MH1 could prey on a wide spectrum of Gram-negative bacteria and a broader range of bacteria, irrespective of phylogeny, in accordance with the common characteristics of *Bdellovibrio* and like organisms and myxobacteria, respectively. Liquid culture assays also found that although predation by *B. stolpii* HI3 rapidly and largely occurred, the prey bacteria regrew, possibly through plastic phenotypic resistance to predation. In contrast, predation by *Myxococcus* sp. MH1 occurred at relatively low efficiency but was longer lasting. The two strains exhibited slightly distinct temperature preferences but commonly preferred slightly alkaline pH. The novel findings of this study provide evidence for the coexistence of predatory bacteria with diverse predation capabilities in the natural aquatic environment.

## 1. Introduction

Predation is a common mode of interaction among organisms. In the microbial world, predation ability is found in bacteriophages, bacteria, protists, and nematodes [1,2]. Predatory bacteria that lyse other bacteria and consume their cell-derived macromolecules as nutrients are ubiquitous in both natural (terrestrial and aquatic) and engineered environments, including freshwater, seawater, soils, plant rhizosphere, sewage, activated sludge in wastewater treatment plants, and human and animal intestines [2]. Recently, there has been mounting evidence on the significant roles of predatory bacteria in microbial food webs and in shaping the microbial community [1,2,3,4]. The bacteriolytic activities of predatory bacteria are effective against a broad range of planktonic and biofilm bacteria and harmless to higher eukaryotic organisms. Because of their unique features, predatory bacteria have attracted increasing attention for various biotechnological applications in the medical, agricultural, aquacultural, and environmental fields, especially as living antibiotics or biocontrol agents [4,5,6,7].

Predatory bacteria are found in various phylogenetic groups, including Proteobacteria, Chloroflexi, Cytophagaceae, and Actinobacteria [8]. Among them, *Bdellovibrio* and like organisms (BALOs) and myxobacteria are the two major categories ubiquitously found in many environments [2,8]. BALOs are a group of small Gram-negative bacteria characterized by obligate predatory feeding behavior; that is, their growth and reproduction entirely depend on their consumption of other bacteria [2,4,8]. They prey on Gram-negative bacteria with an epibiotic or periplasmic predation (direct invasion) strategy [2,4,5]. Over the past two decades, BALOs have attracted increasing attention owing to their potential usefulness in biotechnological applications [2,6,7]. In contrast, myxobacteria are representative facultative predatory bacteria [9]. Myxobacteria are social bacteria that employ a group-hunting strategy for predation and secrete hydrolytic enzymes and secondary metabolites to lyse prey cells [5,9,10,11,12,13]. They are generalists capable of feeding on bacteria (both Gram-negative and -positive bacteria) as well as yeast, fungi, protozoa, and nematodes [5,9,14].

Owing to the increasing interest in the ecological and technological applications of predatory bacteria, the mechanisms and properties of bacterial predation have been studied in detail. However, most current evidence is derived from a limited number of model strains, resulting in a lack of knowledge on the predation characteristics of wild-type environmental predatory bacterial strains [15]. Furthermore, in contrast to extensive studies on the predation of model prey strains or specific pathogenic bacteria in humans, animals, fish, and plants, studies on the predation capabilities of predatory bacteria against diverse nonpathogenic environmental bacteria are still scarce [16,17,18]. Elucidating the predatory relationships between coexisting bacteria will deepen our understanding of the ecological functions of predatory bacteria as well as provide a reference for better biotechnological use of predatory bacteria in the environment.

To address the aforementioned research gaps, this study aimed to obtain novel predatory bacteria from natural freshwater environments and assess their predation capabilities against coexisting phylogenetically diverse environmental bacteria [19]. To this end, we isolated novel predatory bacterial strains from a freshwater pond. Interestingly, the two novel strains isolated were placed in different categories of predatory bacteria, namely BALOs and myxobacteria. Then, the predation properties of the two strains were examined and compared with respect to the prey range against a variety of coexisting bacteria, temporal predation dynamics, predation efficiency, and their responses to temperature and pH as fundamental environmental parameters, hypothesizing that predatory bacteria with different predation capabilities may coexist in natural freshwater environments.

## 2. Materials and Methods

### 2.1. Bacterial Strains

*Escherichia coli* HB101 [20] and 52 environmental bacterial strains (Table S1) were used as potential prey bacteria. *E. coli* has often been used as prey for predatory bacteria [21,22,23]. The 52 environmental strains had been isolated from the same pond water from which the predatory bacteria were isolated, and covered various bacterial taxa, including the phyla Proteobacteria (classes α-, β-, and γ-Proteobacteria), Bacteroidetes (classes Cytophagia and Flavobacteriia), Firmicutes (class Bacilli), and Actinobacteria (class Actinomycetia).

### 2.2. Culture Media and Cultivation Conditions

A double-layer agar plate [24] comprising HM buffer (25 mM HEPES, 3 mM CaCl_2_, and 2 mM MgCl_2_, pH 7.4) supplemented with 1.5% agar at the bottom layer and HM buffer supplemented with the prey and 0.7% agar at the top layer was used to isolate predatory bacteria and culture obligatory predatory strains. R2A broth (DAIGO; Nihon Pharmaceutical, Tokyo, Japan), LB broth (Lennox; Becton, Dickinson and Company, Sparks, MD, USA), and tryptic soy broth (Becton, Dickinson and Company, Sparks, MD, USA) were used to culture the facultative predatory and prey strains. A basal salt medium (BSM; 1 g/L K_2_HPO_4_, 1 g/L (NH_4_)_2_SO_4_, 0.2 g/L MgSO_4_·7H_2_O, 0.05 g/L NaCl, 0.01 g/L CaCl_2_, and 0.01 g/L FeCl_3_, pH 7.4) was used for liquid cultures of the predation assays. Agar was added at 1.5% to prepare the solid media. Unless otherwise noted, cultivation was statically conducted at 28 °C (for a solid medium) or with rotary shaking at 120 rpm (for a liquid medium).

### 2.3. Isolation of Predatory Bacteria

Isolation of predatory bacteria was conducted using *Acidovorax* sp. DW036 and *E. coli* HB101 as prey strains. *Acidovorax* sp. DW036 was a bacterial strain that had been isolated from the same pond water as the predatory bacteria (Table S1). *E. coli* HB101 was chosen because it was considered useful for isolating predatory bacteria based on the evidence that it is often used as prey for various predatory bacteria.

Water samples collected from a freshwater pond in Osaka, Japan (34°82′ N, 135°53′ E), on 14 June and 27 August 2019 were used as microbial sources. The samples were passed through a 10 μm membrane filter (Merck Millipore, Darmstadt, Germany) to remove coarse particles. The filtered samples were co-cultivated with prey to enrich predatory bacteria prior to their isolation. For the June sample, 10 mL of the filtered sample was added to 100 mL LB broth in a 300 mL Erlenmeyer flask in which the prey strain (*Acidovorax* sp. DW036) was precultivated for 1 d. After cultivation for 4 d, the cells in the mixed culture were harvested using centrifugation (20,000× *g*, 4 °C, 35 min) and resuspended in 5 mL HM buffer. In the second trial using the August sample, the cells of the pregrown prey strain (*E. coli* HB101) were added to 20 mL of the filtered water sample in a 50 mL glass vial to obtain an optical density at the wavelength of 600 nm (OD_600_) of 10. After 4 d of cultivation, the mixed culture with a large OD_600_ reduction was used for the isolation step. OD_600_ was measured using a UV-1700 UV–vis spectrophotometer (Shimadzu, Kyoto, Japan).

A double-layer agar plating technique was applied to isolate predatory bacteria from the pretreated mixed cultures. *Acidovorax* sp. DW036 and *E. coli* HB101 (Table S1) were used as prey for the June and August samples, respectively. The prey bacteria were cultured in LB broth for 1 d, following which the cells were harvested by centrifugation (10,000× *g*, 4 °C, 5 min), washed twice with HM buffer, and resuspended in HM buffer to obtain an OD_600_ of 50. The prepared prey solution (300 μL) and serial dilutions of the pretreated sample (100 μL) were mixed with the top layer (5 mL) of a double-layer agar plate. The plates were incubated for 3–5 d until lytic halos (clear zones caused by lysis of the prey) appeared. The agar plate segment with a lytic halo was collected, homogenized, and re-inoculated into a fresh double-layer agar plate. This procedure was repeated to obtain pure predatory bacterial strains.

### 2.4. Phylogenetic Identification

The isolated predatory bacteria were identified based on 16S ribosomal RNA (rRNA) gene sequences. The partial 16S rRNA gene sequences were amplified using primers 27F and 1392R [25,26], and then sequenced by Macrogen Japan (Kyoto, Japan). Phylogenetic analyses of the sequences of the isolated strains and their relatives, which were identified using a BLAST search (http://www.ncbi.gov/blast/, accessed on 2 January 2022), were conducted using MEGA version X [27].

### 2.5. Prey Range Determination

The prey range of the isolated predatory bacteria was determined using the double-layer agar plating technique. Fifty-three strains were used as prey, including *E. coli* HB101 and 52 strains that had been isolated from the same pond as the predatory bacteria (Table S1). The prey was precultivated in R2A broth for 1 d, following which the cells were harvested, washed, and mixed with the top layer medium of the double-layer agar plate (OD_600_ = 50). For the obligate predatory strain, the precultured agar plate segment with a lytic halo was collected, homogenized, and mixed with HM buffer into which *E. coli* HB101 was inoculated (OD_600_ = 10). After cultivation for 1 d, the culture was filtered through a 0.45 μm membrane filter (Advantec, Tokyo, Japan) three times to remove the prey, following which the filtrate (5 μL) was spotted onto the prepared double-layer agar plate. For the facultative predatory strain, a colony formed on the LB plate was picked up and stabbed on the prepared double-layer agar plate using an inoculating needle. Each plate was incubated for 2 weeks and examined for the formation of a lytic halo. Each experiment was performed in triplicate, with a positive control using *E. coli* HB101 as the prey and a negative control without the inoculation of any predator.

### 2.6. Liquid Culture Predation Assay

Predation assays in liquid cultures using *E. coli* HB101 as prey were performed to (1) monitor predator–prey dynamics in detail and (2) examine the effects of temperature and pH on predation. The assays under each condition were performed in triplicate, unless otherwise noted.

Prior to the predation assay, the prey and facultative predatory strains were precultivated in R2A and LB broth, respectively, for 1 d. The obligate predator was precultivated in HM buffer inoculated with *E. coli* HB101 as the prey for 2 d, following which the resultant culture was filtered through a 0.45 μm filter twice to remove the prey. Cells in each culture were harvested, washed once with BSM, and inoculated into fresh BSM. The initial OD_600_ value of the prey was set to 1.

The first series of liquid culture predation assays were conducted in 100 mL BSM in a 300 mL baffled Erlenmeyer flask for 7 d. Temporal variations in predatory and prey strains were monitored using a viability quantitative polymerase chain reaction (qPCR). Moreover, the dissolved organic carbon (DOC) concentration was measured using a total organic carbon analyzer (TOC-VCSH, Shimadzu) after filtration through a 0.2 µm filter (Advantec).

The second assay series was conducted in 30 mL of BSM in a 50 mL glass vial for 3 d, following which temporal variations in the prey strain were monitored. The effect of temperature was tested at 15, 20, 25, 30, 37, and 42 °C, with an initial pH of 7.4, whereas that of pH was examined by adjusting the initial pH to 5.6, 6.2, 6.8, 7.4, 8.0, and 8.6, at a consistent temperature of 28 °C.

### 2.7. Viability qPCR

Viability qPCR was used to enumerate the viable cells of predatory and prey bacteria. The samples were pretreated with PMAxx prior to DNA extraction. PMAxx is an improved propidium monoazide that can cross the cell membranes of dead or damaged cells and irreversibly intercalate into their DNA upon photoactivation, thereby interfering with PCR amplification [28]. PMAxx (Biotium, Fremont, CA, USA) was added to the culture sample (200 µL) at a concentration of 10 µM, following which the sample was incubated in the dark at room temperature for 10 min and then exposed to light in an LED Crosslinker (Takara Bio, Shiga, Japan) for 15 min. The sample was then centrifuged (20,000× *g*, 4 °C, 5 min), and the pellet was washed with ultrapure water for DNA extraction using a Cica Geneus DNA extraction kit (Kanto Chemical, Tokyo, Japan).

qPCR was performed using strain-specific primers. Primers for the isolated predatory bacteria were designed using the Primer-BLAST program [29] to specifically amplify the 16S rRNA gene of each of the target strains and attain a high amplification efficiency (>80%) in our qPCR system (Table 1). In addition, previously designed primers targeting the *tbpA* gene [30] were used for the prey (*E. coli* HB101) (Table 1). The qPCR analysis was conducted with a CFX Connect Real-Time PCR Detection System (Bio-Rad, Hercules, CA, USA) using GeneAce SYBR qPCR Mix α (Nippon Gene, Tokyo, Japan) and 0.2 µM each of the forward and reverse primers. The thermal cycling conditions were as follows: 50 °C for 2 min, 95 °C for 10 min, and 40 cycles of 95 °C for 30 s and 60 °C, 65 °C, or 66 °C (Table 1) for 1 min. The optimal thermal cycling conditions for the isolated predatory bacteria were individually determined by preliminary experiments with different annealing and extension temperatures. Standard DNA to quantify each target gene was prepared as previously described [31], with minor modifications. The DNA copies obtained using qPCR were converted to cell numbers based on the copy number of each gene in the genome of the host strain.

## 3. Results and Discussion

### 3.1. Isolation and Identification of Two Predatory Bacterial Strains

The formation of transparent and round lytic halos was successfully observed on the double-layer plates in both attempts to isolate predatory strains from pond water: the first attempt using the June sample and *Acidovorax* sp. DW036, and the second attempt using the August sample and *E. coli* HB101. After repetitive purifications, one predatory strain was successfully isolated in each attempt. The strain isolated in the first attempt, named MH1, formed a lytic halo with a fruiting body (Figure S1b). The other strain isolated in the second attempt was named strain HI3, and this strain formed plaques without fruiting bodies (Figure S1a).

**Figure 1:**
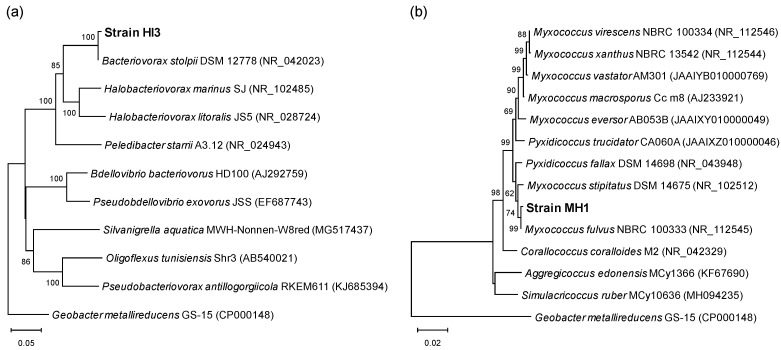
Phylogenetic tree based on 16S rRNA gene sequences from isolated predatory strains HI3 (**a**) and MH1 (**b**) and the type strains of their related genera. Evolutionary relationship was inferred using neighbor-joining method, with Kimura 2-parameter model. *Geobacter metallireducens* GS-15^T^ was used as an outgroup. Accession numbers of nucleotide sequences for type strains are provided in parentheses. Bootstrap values >50% based on 1000 replicates are shown at nodes. Scale bar represents 0.05 (**a**) or 0.02 (**b**) nucleotide substitution per site.

Based on a BLAST search, the 16S rRNA gene sequences of strains HI3 (1269 bp; LC505636) and MH1 (1295 bp; LC506125) were closely related to those of the genus *Bacteriovorax* and the family Myxococcaceae, respectively. The results of further phylogenetic analysis revealed that strain HI3 had the highest sequence identity (99.84%) with *Bacteriovorax stolpii* DSM 12778T (NR_042023), whereas strain MH1 showed the highest sequence identity of 99.92% with *Myxococcus fulvus* NBRC 100333T (NR_112545), followed by 99.38% with *Myxococcus stipitatus* DSM 14675T (NR_102512) (Figure 1). Therefore, the novel strains HI3 and MH1 were identified as *B. stolpii* and *Myxococcus* sp., respectively.

Only strain MH1 grew on nutrient media, including R2A, TSB, and LB, in addition to the double-layer agar plate containing prey bacteria. This facultative predation and the aforementioned fruiting body formation of strain MH1 were also consistent with the common features of myxobacteria [9,10,11,12,13].

Surprisingly, in spite of the successful isolation of a *Bacteriovorax* strain, no *Bdellovibrio* or *Peredibacter* strains, which may be similarly or more abundant in freshwater environments [2], were isolated in this study. Although the specific cause could not be clarified in this study, the following were raised as the possible reasons: (1) *Bacteriovorax* was more abundant than *Bdellovibrio* or *Peredibacter* in the isolation source used in this study; (2) the cultivation conditions applied in this study might be preferable for the growth of *Bacteriovorax*; and (3) *Bdellovibrio* or *Peredibacter* might have been lost during the isolation procedure.

The coexistence of various predatory bacteria has been previously observed [1,3,12]. However, to the best of our knowledge, no study has simultaneously evaluated the predation properties of predatory bacteria within distinct categories (e.g., BALOs and myxobacteria) under the same environmental conditions. Thus, our characterization of the two distinct predatory bacterial strains isolated herein can be considered meaningful for expanding our knowledge on predation by environmental predatory bacteria.

### 3.2. Prey Range of Isolated Strains

To determine the prey range of the two isolated predators, screening experiments were conducted with 52 potential prey strains isolated from pond water, as well as an *E. coli* strain.

**Figure 2:**
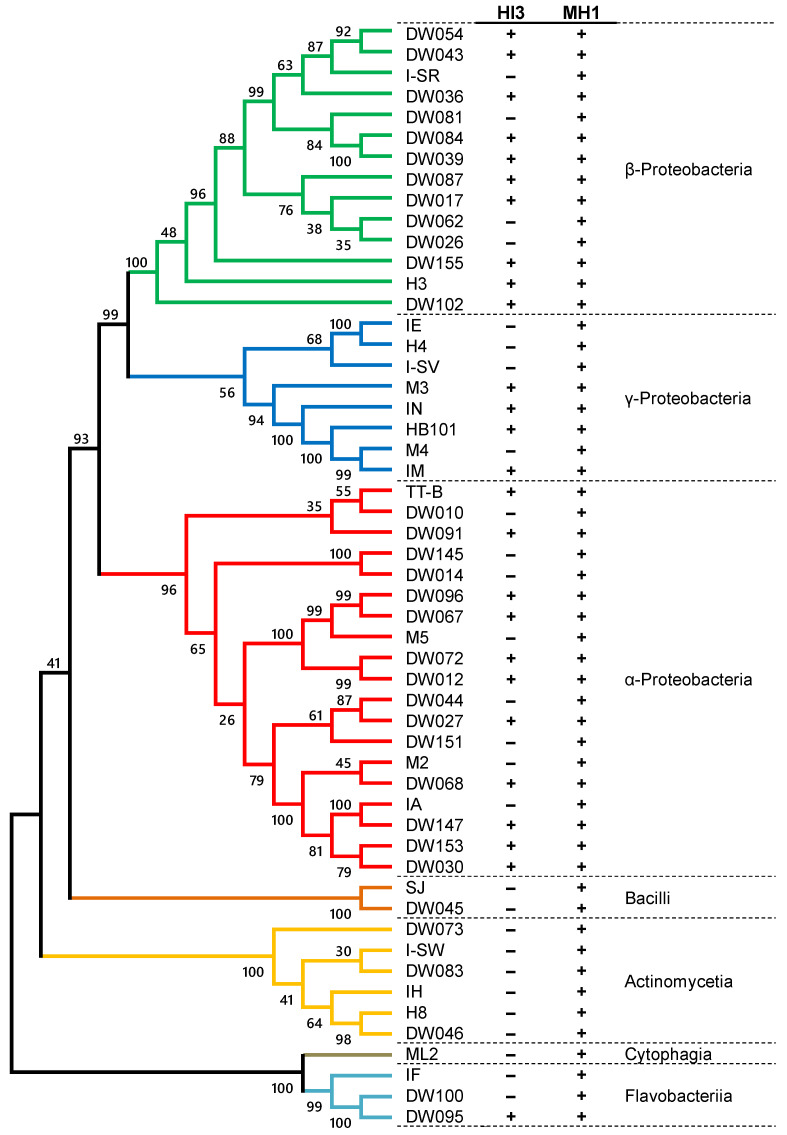
Prey range specificities of *Bacteriovorax stolpii* HI3 and *Myxococcus* sp. MH1 on 53 potential prey strains. Detailed phylogenetic information of potential prey strains is provided in Table S1. +, predation; −, no predation.

*B. stolpii* HI3 did not form a visible plaque on any of the eight Gram-positive strains, but it preyed on 26 out of the 45 Gram-negative strains (Figure 2). The susceptible Gram-negative strains included α-, β-, and γ-Proteobacteria and Flavobacteriia. Notably, in the predation of all susceptible strains by strain HI3, visible plaques were formed within 4 d, and multiple plaques were found around the portion where the predator was spotted. The ability to prey on a broad array of Gram-negative bacteria but not Gram-positive bacteria is consistent with the typical features of BALOs [8,15]. However, while predation of phylogenetically diverse Gram-negative bacteria has been experimentally demonstrated in *Bdellovibrio* strains [17,32,33], this is the first study to demonstrate that *Bacteriovorax* is capable of lysing a broad range of Gram-negative bacteria, including Flavobacteriia. The susceptibility of different strains in the same genus to predation by strain HI3 was the same in most cases: commonly susceptible in *Acidovorax* (DW036, DW039, and DW084) and *Novosphingobium* (DW067 and DW096); and commonly tolerable in *Asticcacaulis* (DW014 and DW145), *Ideonella* (DW026 and DW062), and *Stenotrophomonas* (H4 and IE). However, between the two *Ensifer* strains, DW068 was susceptible, whereas M2 was tolerant to predation by strain HI3. Distinct predation susceptibility of strains belonging to the same genus or species has been found in certain BALO strains, including *Bdellovibrio*, *Bacteriovorax*, and *Micavibrio* [32,33,34,35]. It has been suggested that the host specificity of a BALO strain is determined by the congeniality between the predation mechanisms of the BALO strain (e.g., strategy, lytic enzymes, etc.) and the species- and strain-specific defense mechanisms of the prey strain (e.g., cell wall composition) [2,5]. Further studies are required to determine the significant determinants of host specificity.

In an assay employing *Myxococcus* sp. MH1 as a predator, lytic halos were formed for all 53 potential prey strains (Figure 2). These results demonstrated that strain MH1 is capable of attacking and preying on both Gram-negative and -positive bacteria. This is in line with previous evidence on the phylogenetically broad prey spectrum of *Myxococcus* spp. [1,14,36]. In contrast, predation efficiency varied depending on prey strain. While lytic halos appeared within 3 days in most cases, the formation of visible halos required more than 1 week in several prey strains. Particularly, strains DW045 (*Bacillus* in Firmicutes), IA (*Beijerinckia* in Proteobacteria), I-SW (*Nakamurella* in Actinobacteria), ML2 (*Dyadobacter* in Bacteroidetes), and SJ (*Staphylococcus* in Firmicutes) appeared to be relatively tolerant to predation by strain MH1. These results corroborate previous findings on the relative preference of *Myxococcus* spp. for Gram-negative strains over Gram-positive strains [1,14,36].

### 3.3. Temporal Dynamics of Predation by the Isolated Strains

To determine predation by strains HI3 and MH1 over time, predation assays were conducted in liquid culture using *E. coli* HB101 as the model prey. Previous studies have mentioned the possibility of inaccurate counting of individual bacterial species using culture-based analysis [15,37], and that predatory bacteria can prey on even viable but nonculturable cells [38]. In addition, we confirmed that during co-cultivation with HI3 and HB101, the viability qPCR could provide similar reduction trends of HB101 to those of the culture-based method, with a higher sensitivity (Figure S2). Therefore, this study applied viability qPCR for accurate quantitative monitoring of predation dynamics.

**Figure 3:**
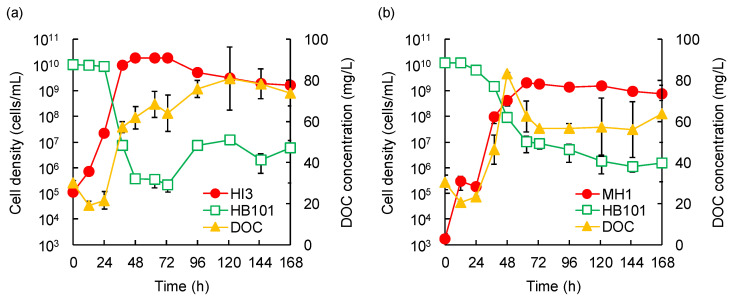
Temporal changes in predator and prey cell densities and dissolved organic carbon (DOC) concentration, during co-cultivation of *Bacteriovorax stolpii* HI3 (**a**) or *Myxococcus* sp. MH1 (**b**) as predator, with *Escherichia coli* HB101 as prey. Error bars represent standard deviation (*n* = 3).

Without predators, the cell density of HB101 remained stable at approximately 10^10^ cells/mL until 168 h (Figure S3). In contrast, when HB101 was co-cultured with HI3 or MH1, its cell density notably declined, together with the proliferation of predators and increased DOC concentration, which was indicative of prey lysis (Figure 3). However, the temporal trends of predation differed depending on the predatory strain.

In the co-culture of HI3 and HB101, the cell density of HB101 remained stable at nearly 10^10^ cells/mL until 24 h, after which it drastically declined to 3.7 × 10^5^ cells/mL at 48 h and almost stabilized thereafter (Figure 3a). Along with the decrease in HB101, DOC concentration rapidly increased from 21.6 mg/L at 24 h to 57.3 mg/L at 38 h. Moreover, the cell density of HI3 drastically increased from 1.1 × 10^5^ cells/mL at 0 h to nearly 10^10^ cells/mL at 38 h. These results clearly indicated the host-dependent growth of HI3 on HB101. After 72 h, the cell density of HI3 gradually decreased to nearly 10^9^ cells/mL, until 168 h, together with a slight increase in DOC concentration, which suggested partial death of HI3 owing to the lack of sufficient predation. In contrast, HB101 cell density increased after 72 h, reaching approximately 10^7^ cells/mL. This increase likely resulted from the utilization of solubilized cell components released by the initial predation of HB101 by HI3 and subsequent death of HI3.

When HB101 was co-cultured with MH1, the density of HB101 (1.2 × 10^10^ cells/mL at the beginning of experiments) started decreasing after 12 h and reached 1.0 × 10^7^ cells/mL at 63 h (Figure 3b). Concomitantly, the density of MH1 increased from 1.7 × 10^3^ cells/mL at 0 h to 2.0 × 10^9^ cells/mL at 63 h. Furthermore, DOC concentration increased from 20.5 mg/L at 12 h to 83.3 mg/L at 48 h; however, it again decreased to 62.5 mg/L at 63 h. These results indicated the predation of HB101 by MH1. Moreover, the decline in DOC concentration from 48 to 63 h was possibly caused by the partial utilization of solubilized HB101 cell components by MH1, which can also independently grow from predation. After 63 h, MH1 remained stable until 168 h, with only a slight decrease over time. In contrast, the density of HB101 continuously declined to nearly 10^6^ cells/mL and never increased until 168 h.

In predation by both HI3 and MH1, the decrease in HB101 cells markedly stopped or slowed after a rapid decline, and HB101 was not completely eradicated (Figure 3). Similar phenomena have been observed in predation assays using different predators, such as *Bdellovibrio* [17,39] and *Micavibrio* [32], suggesting that this may be a common property of predation between bacteria, irrespective of the predator phylogeny, including BALOs and myxobacteria. Furthermore, Shemesh and Jurkevitch [40] observed no total elimination or regrowth of prey cells in the co-culture of several combinations of predators (BALOs) and prey, as found in the co-culture of HB101 and HI3 in this study (Figure 3a). This indicates that the incomplete eradication of prey cells does not stem from the incapability of the predator to attack low concentrations of prey cells but from the inherent plastic phenotypic resistance of prey populations to predation, which is not unique to specific predators or prey [40].

Between the two predators, the decrease in HB101 cell density was larger and more rapid in predation by HI3 than in that by MH1 (Figure 3). To the best of our knowledge, there are no reports on the direct comparison of predation efficiencies between predators belonging to BALOs and myxobacteria. The detailed reasons for the difference in predation efficiencies between HI3 and MH1 could not be identified in the present study, although it is likely affected by their distinct growth properties, namely obligate and facultative predation, respectively. In contrast, the decrease in HB101 cells lasted longer in predation by MH1, albeit at a low efficiency (Figure 3b). This would also be a characteristic feature of predation by myxobacteria, which lyse prey cells by secreting lytic enzymes.

### 3.4. Effects of Temperature and pH on the Predation Activities of the Isolated Strains

As fundamental environmental factors, the effects of temperature (15–42 °C) and pH (5.6–8.6) on the predation activities of the isolated strains were assessed by predation assays in liquid culture using *E. coli* HB101 as the model prey. The decline in HB101 cell density was monitored using viability qPCR.

**Figure 4:**
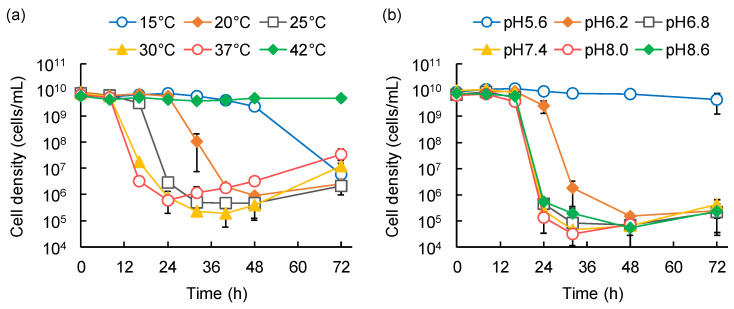
Effects of temperature (**a**) and pH (**b**) on *Bacteriovorax stolpii* HI3 predation on *Escherichia coli* HB101. Temporal variations of HB101 cell density were monitored. Error bars represent standard deviation (*n* = 3, except several data where *n* = 2).

Strain HI3 decreased the cell density of HB101 by three to five orders of magnitude, at 15–37 °C and pH 6.2–8.6, but not at 42 °C and pH 5.6, within 72 h of the experimental period (Figure 4). Between 15 °C and 30 °C, the lag period to initiate HB101 decline was shortened with an increase in temperature (Figure 4a). From 30 °C to 37 °C, although the length of the lag period was the same, the decline in HB101 cell density was slightly faster at 37 °C than that at 30 °C. Regarding pH, the lag period and overall lysis efficiency in predation by HI3 were similarly effective at pH 6.8–8.6, although the most effective pH was 8.0 (Figure 4b). These results suggested that conditions of approximately 30–37 °C and neutral to slightly alkaline pH are suitable for predation by HI3. Reportedly, *Bacteriovorax* spp. can grow at 15–35 °C, and the optimum growth temperature for *B. stolpii* Uki2^T^ is 28–30 °C [41,42]. It has also been reported that predation of *Vibrio parahaemolyticus* by *Bacteriovorax* sp. BV-A most efficiently occurs at 30 °C and pH 7–8 [35]. Therefore, both the optimum temperature and pH suggested for predation by HI3 seem to be similar or slightly higher than those reported for *Bacteriovorax* spp.

**Figure 5:**
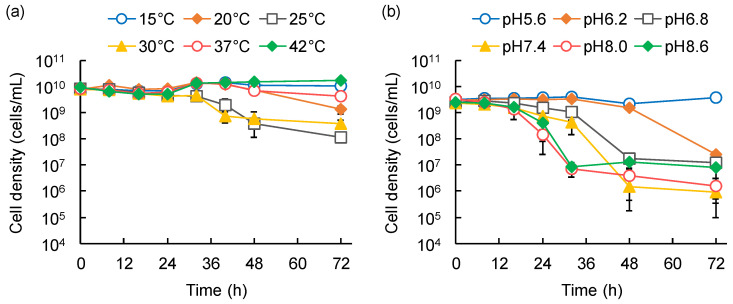
Effects of temperature (**a**) and pH (**b**) on *Myxococcus* sp. MH1 predation on *Escherichia coli* HB101. Temporal variations of HB101 cell density were monitored. Error bars represent standard deviation (*n* = 3, except several data where *n* = 2).

Among the temperatures tested, HB101 cell density notably declined due to predation of MH1 at 20–37 °C, with the most effective lysis (a decline of one order of magnitude within 40–48 h) observed at 25–30 °C (Figure 5a). No detectable decline in HB101 was observed at 15 °C and 42 °C. In contrast, among the examined pH ranges, the decline in HB101 was observed at pH 6.2–8.6, with the most effective lysis (a decline of two orders of magnitude within 32 h) at pH 8.0–8.6 (Figure 5b). The lysis efficiency of MH1 decreased with pH reduction at below pH 8.0. Therefore, predation by MH1 was effective at 25–30 °C and pH 8.0–8.6, and appeared to be sensitive to temperature, as compared with the predation by HI3. Thus, the optimum temperature for predation by MH1 was similar to that reported for *M. fluvus* strains (26–32 °C), but the optimum pH was slightly alkaline (pH 6.8–7.6 for *M. fluvus*) [43,44]. The slight differences might be affected by the distinct assay systems; this study assessed predation-dependent growth with the lysis of prey strains as an indicator, whereas other studies evaluated the growth on nutrient media.

## 4. Conclusions

In this study, we isolated two predatory bacterial strains allocated to distinct categories (i.e., BALOs and myxobacteria) from a freshwater pond and characterized their predation capabilities. *B. stolpii* HI3 and *Myxococcus* sp. MH1 isolated in this study had common prey ranges for BALOs and myxobacteria, respectively, against environmental bacteria. The two predators also differed, especially in terms of the efficiency and durability of hydrolysis and predation activity. These results provide direct evidence that predatory bacteria with distinct predation capabilities coexist in natural aquatic environments. This might also imply that multiple predatory bacteria intricately and cooperatively shape the structure of microbial communities in certain environments, although further study is needed to confirm the hypothesis.

However, the knowledge obtained in this study was limited to the differences in the fundamental characteristics of the two predatory bacterial strains. Further study including molecular analysis is needed for in-depth characterization of their predation capabilities. In contrast, given the diversity of predatory bacteria in the environment, further exploration of the functional diversity of predatory bacteria, including those within the same and different categories, in certain environments will be interesting. Furthermore, in addition to the predation mechanisms of a predatory strain, how multiple predatory bacteria collaborate to shape a microbial community is also an important topic to be addressed to decipher the ecological functions of predatory bacteria.

Despite the limited knowledge, our results made aware of the importance of selecting appropriate predatory strains depending on the situation, including target bacterial species, necessary predation efficiency and duration, and environmental conditions, in the biotechnological use of predatory bacteria. Further exploration of diverse predatory bacterial strains will be helpful for various application situations.

## Figures and Tables

**Table 1:** Primers used for qPCR analysis.

Strain	Sequence (5′–3′)	Products of the Targeted Genes	Annealing and Extension Temp. (°C)	Amplicon Size (bp)	Amplification Efficiency (%)	Correlation Coefficient (r^2^)	Reference
HI3	TAAACGAGGGAGTGCCCTTC	16S rRNA	60	160	88.2–92.6	0.994–0.999	This study
	GTTAGCCCAGGCAGTCTTTCTA						
MH1	ACGAAAACCCGTAGCCCAAC	16S rRNA	65	160	82.5–93.4	0.992–1.000	This study
	TTCACACCCGACTTGTCACG						
HB101	GCATCCATAGCAACAGACCCA	Thiamine-binding periplasmic protein	66	105	81.9–92.7	0.990–0.999	[30]
	CGCCACAAAGCCTGAAAGAA						

## Data Availability

The partial 16S rRNA gene sequences obtained in this study have been deposited in the GenBank/EMBL/DDBJ databases under accession numbers LC505636 (*B. stolpii* HI3), LC506125 (*Myxococcus* sp. MH1), and LC701001–LC701034 (potential prey strains).

## Supplementary Materials

The following supporting information can be downloaded at: https://www.mdpi.com/article/10.3390/microorganisms10091816/s1, Table S1: List of potential prey strains used in this study; Figure S1: Lytic halos formed by isolated predatory strains on double-layer agar plates; Figure S2: Temporal changes in predator and prey cell densities during co-cultivation of *Bacteriovorax stolpii* (predator) and *Escherichia coli* HB101 (prey) that were quantified by viability qPCR and culturing method; Figure S3: Temporal changes in *Escherichia coli* HB101 cell density and DOC concentration in a single culture without inoculation with any predatory strains.
