# Supplementary material for: Characterization of Two Novel Predatory Bacteria, Bacteriovorax stolpii HI3 and Myxococcus sp. MH1, Isolated from a Freshwater Pond: Prey Range, and Predatory Dynamics and Efficiency
Source: Microorganisms. 2022 Sep 10;10(9):1816. doi: 10.3390/microorganisms10091816 (PMC9505378; doi:10.3390/microorganisms10091816)
Supplement: Supplementary file 1 [file microorganisms-10-01816-s001.zip › microorganisms-1904735-supplementary.pdf]

Table S1. List of potential prey strains used in this study

Strain	Order	Genus	16S rRNA gene accession no.
DW010	α-Proteobacteria	*Roseomonas*	LC701001
DW012	α-Proteobacteria	*Rhizorhabdus*	LC573444
DW014	α-Proteobacteria	*Asticcacaulis*	LC573430
DW017	β-Proteobacteria	*Aquincola*	LC701002
DW026	β-Proteobacteria	*Ideonella*	LC701003
DW027	α-Proteobacteria	Phreatobacteraceae*	LC701004
DW030	α-Proteobacteria	*Rhizobium*	LC701005
DW036	β-Proteobacteria	*Acidovorax*	LC701006
DW039	β-Proteobacteria	*Acidovorax*	LC573433
DW043	β-Proteobacteria	*Curvibacter*	LC573434
DW044	α-Proteobacteria	*Azorhizobium*	LC701007
DW045	Bacilli	*Bacillus*	LC701008
DW046	Actinomycetia	*Microbacterium*	LC701009
DW054	β-Proteobacteria	*Rhodoferax*	LC701010
DW062	β-Proteobacteria	*Ideonella*	LC701011
DW067	α-Proteobacteria	*Novosphingobium*	LC573443
DW068	α-Proteobacteria	*Ensifer*	LC701012
DW072	α-Proteobacteria	*Sphingomonas*	LC701013
DW073	Actinomycetia	*Mycobacterium*	LC701014
DW081	β-Proteobacteria	*Pseudacidovorax*	LC573435
DW083	Actinomycetia	*Nocardioides*	LC701015
DW084	β-Proteobacteria	*Acidovorax*	LC701016
DW087	β-Proteobacteria	*Pelomonas*	LC701017
DW091	α-Proteobacteria	*Niveispirillum*	LC701018
DW095	Flavobacteriia	*Epilithonimonas*	LC701019
DW096	α-Proteobacteria	*Novosphingobium*	LC573445
DW100	Flavobacteriia	*Chryseobacterium*	LC573436
DW102	β-Proteobacteria	*Methylophilus*	LC573439
DW145	α-Proteobacteria	*Asticcacaulis*	LC573432
DW147	α-Proteobacteria	*Agrobacterium*	LC701020
DW151	α-Proteobacteria	Beijerinckiaceae*	LC701021
DW153	α-Proteobacteria	*Peteryoungia*	LC701022
DW155	β-Proteobacteria	*Herbaspirillum*	LC573441
H3	β-Proteobacteria	*Aquitalea*	LC191967
H4	γ-Proteobacteria	*Stenotrophomonas*	LC191968
H8	Actinomycetia	*Leucobacter*	LC191972
HB101	γ-Proteobacteria	*Escherichia*	AP009048
IA	α-Proteobacteria	*Beijerinckia*	LC701023
IE	γ-Proteobacteria	*Stenotrophomonas*	LC701024
IF	Flavobacteriia	*Flavobacterium*	LC701025
IH	Actinomycetia	*Kocuria*	LC701026
IM	γ-Proteobacteria	*Citrobacter*	LC701027
IN	γ-Proteobacteria	*Aeromonas*	LC701028
I-SR	β-Proteobacteria	*Caenimonas*	LC701029
I-SV	γ-Proteobacteria	*Hydrocarboniphaga*	LC701030
I-SW	Actinomycetia	*Nakamurella*	LC701031

M2	α-Proteobacteria	*Ensifer*	LC191976
M3	γ-Proteobacteria	*Acinetobacter*	LC191977
M4	γ-Proteobacteria	*Enterobacter*	LC191978
M5	α-Proteobacteria	*Blastomonas*	LC191979
ML2	Cytophagia	*Dyadobacter*	LC701032
SJ	Bacilli	*Staphylococcus*	LC701033
TT-B	α-Proteobacteria	*Reyranella*	LC701034

*The family name is provided because the genus has not been identified.

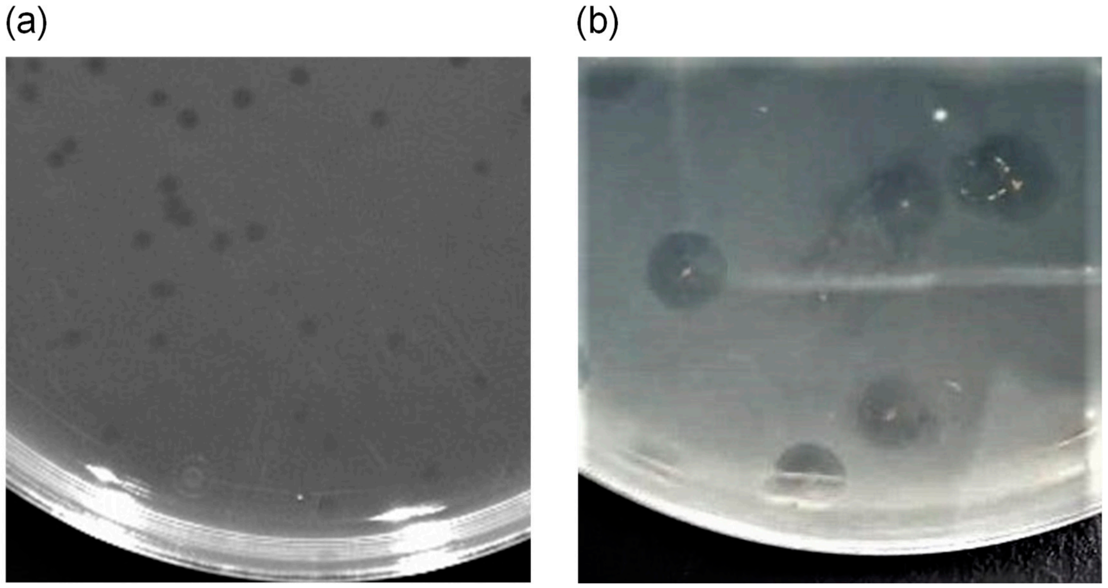

Figure S1. Lytic halos formed by isolated predatory strains on double-layer agar plates. (a), strain HI3 (without fruiting body formation); (b), strain MH1 (with fruiting body formation at the center of the halo).

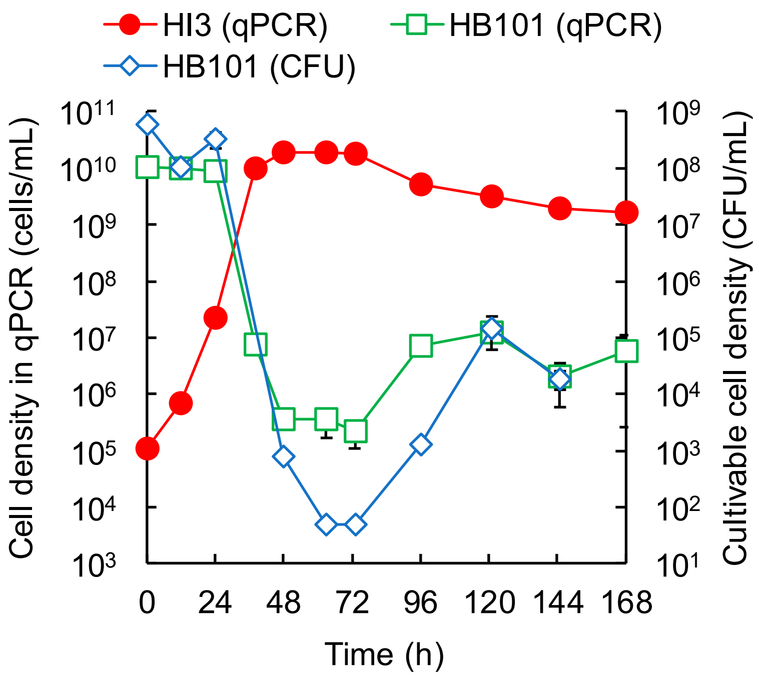

Figure S2. Temporal changes in predator and prey cell densities during co-cultivation of *Bacteriovorax stolpii* (predator) and *Escherichia coli* HB101 (prey). *E. coli* HB101 cell densities were quantified by viability qPCR and culturing method using an R2A agar medium. Error bars represent standard deviation (*n*=3).

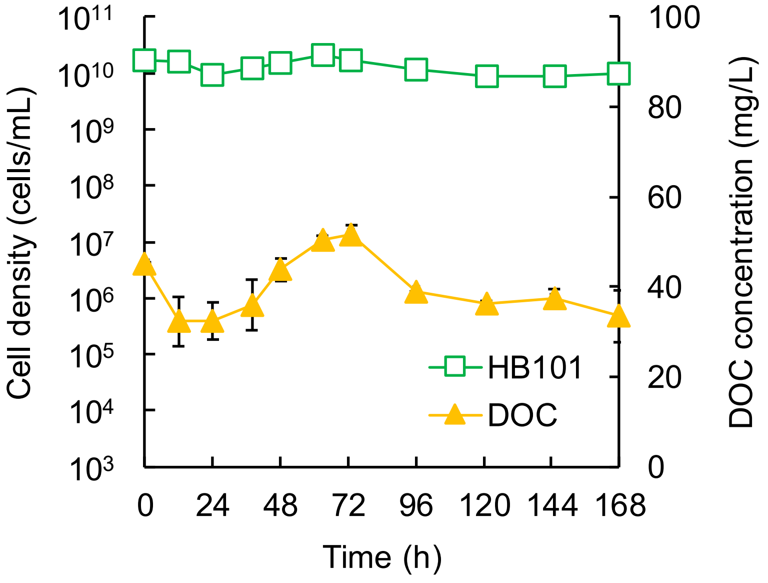

Figure S3. Temporal changes in *Escherichia coli* HB101 cell density and DOC concentration in a single culture without inoculation with any predatory strains. Error bars represent standard deviation (n=3).
